# Direct measurement of malrotation of traumatic femoral neck fractures after osteosynthesis: Introduction of a novel method and interrater reliability

**DOI:** 10.1371/journal.pone.0250409

**Published:** 2021-04-26

**Authors:** Tarek Omar Pacha, Lena Sonnow, Gesa Helen Poehler, Tilman Graulich, Mohamed Omar, Timo Stubig, Christian Krettek, Emmanouil Liodakis

**Affiliations:** 1 Trauma Department, Hannover Medical School (MHH), Lower Saxony, Germany; 2 Department of Radiology, Hannover Medical School (MHH), Lower Saxony, Germany; University Hospital Zurich, SWITZERLAND

## Abstract

**Background:**

In elderly patients, displaced femoral neck fractures are mostly treated by arthroplasty; however for younger patients (<50 years), open reduction and internal fixation is considered the gold standard approach. Despite there being no consensus on the specific procedure, everyday clinical practice in a level I trauma center has shown that postoperative maltorsion after internal fixation of femoral neck fractures can have a significantly worse impact on mobilization and outcome. Different methods for measurement of malrotations are reported in literature. However, any reported method for the assessment of a shaft malrotation in the femur does not work here. In femoral neck fractures, the pointer function of the femoral neck, which is absolutely essential for these techniques, is lost and cannot be set in relation to the condylar plane. These circumstances are not addressed in literature thus far. Therefore, we propose here a novel method to fill this diagnostic gap.

**Methods and findings:**

Three investigators (1 orthopaedic surgeons and 2 radiologists) measured the torsion of 20 legs on 10 patients using the Jarret method and a new geometric technique. To determine the intraobserver reliability the torsional angles were calculated again after 3 months. We applied a new geometric technique, without the need to include the femoral condyles in the measurement, to directly measure the angulation. For torsional difference, the interrater reliability -ICC (interclass correlation) between all investigators was 0.887 (good) (significance level: 95%CI, 0.668–0.969; p<0.001), by using the method of Jarret et al. and 0.933 (good) for the novel technique (significance level: 95%CI, 0.802–0.982; p<0.001). If the examinations are classified according to the patient side, our data show that for established methods, an ICC between the examiners on the right lower extremity is 0.978 (good) (95%CI, 0.936–0.994; p<0.001) and that on the left extremity is 0.955 (good) (95%CI, 0.867–0.988; p<0.001). Comparing with the new method, the right side assumes an ICC of 0.971 (good) (95%CI, 0.914–0.992; p<0.001), while the left side assumes an ICC of 0.910 (good) (95%CI, 0,736–0.976; p<0.001). When it comes to the intraobserver reliability, the measured cohort shows a significant better ICC for the novel method compared to Jarrett et al, with 0.907 respectively 0.786 for comparison in torsional differences.

**Conclusion:**

The established methods may fail in assessing this special aspect of malrotation after femoral neck fractures. Here, the method presented results in a significant difference between the injured and uninjured side and shows significant differences in results compared to conventional measurement methods. The inter- and intraobserver reliability determined in this study is excellent and even higher in the assessment of torsional differences than the established method. We believe that the measurement method presented in this study is a useful tool to objectify the postoperative deformities in this area and making therapy recommendations in the future.

## Introduction

Torsional malalignment after shaft fractures of long bones is a very well-known clinical complication that can affect both the lower as well as the upper extremity [1–7].

The incidence of postoperative maltorsion greater than 10° after femoral nailing is around 41.7% [8, 9]. Upon analyzing 220 femoral shaft fractures treated in our institution, we previously found the incidence of maltorsion>10° to be 43.2% and maltorsion>15° to be 22.7% [7].

Following literature recommendation, differences up to 15° malrotation needs no corrective intervention s [1, 10, 11]. Most often, the malrotation occurs in the shaft of the femur [8] but is also a problem in patients with proximal femoral and femoral neck fractures [12].

To the best of our knowledge, torsional malalignment after femoral neck fractures has not been previously described. In elderly patients, for displaced femoral neck fractures the treatment of choice is the arthroplasty [13]; however, for younger patients (<50 years), open reduction and internal fixation is considered the gold standard approach, although there is no consensus on the preferred specific procedure (cannulated screw fixation vs. sliding hip screw) [14, 15].

The reported high rate of avascular necrosis, nonunion, malunion, and implant failure in young patients [15] as well as the common practice of providing joint replacement for older patients [13] may be the reason for the rare reporting of this complication after osteosynthesis in case of a fractured femoral neck.

However, everyday clinical practice in a level I trauma center shows that postoperative maltorsion after internal fixation of femoral neck fractures can have a significant impact on mobilization and outcome.

While the general incidence of hip fractures is increasing because of demographic changes [16], the number of high energy fractures in young patients is not affected [14, 16, 17].

While postoperative diagnosis of malrotation in adults is usually done by computed tomography (CT) [1, 12, 18], intraoperative detection of malrotation in long bone fractures is difficult. In literature, many suggestions are made without considering the impact of the general incidence of malrotation [1, 19–21].

As for postoperative diagnosis of malrotation, CT is the method of choice [12], although several methods of measuring are reported [10, 12, 22]. A widely used technique was reported by Jarrett et al. [10, 22].

Due to this method being validated for fractures distal to the femoral neck and proximal to the femoral condyles, the application of this method in fractures proximal to the trochanter plane is not reported in literature.

Closer inspection of the anatomic landmarks of the femoral neck reveals that this method and any other reported method for the assessment of a shaft malrotation in the femur may not work. In femoral neck fractures, the pointer function of the femoral neck, which is absolutely essential for this technique, is lost and cannot be set in relation to the condylar plane.

Therefore, the aim of this retrospective study was to report and describe a novel technique for measurement of fractures in the femoral neck plane to reliably and objectively display the torsional error that has remained after osteosynthesis.

## Materials and methods

**Fig 1:**
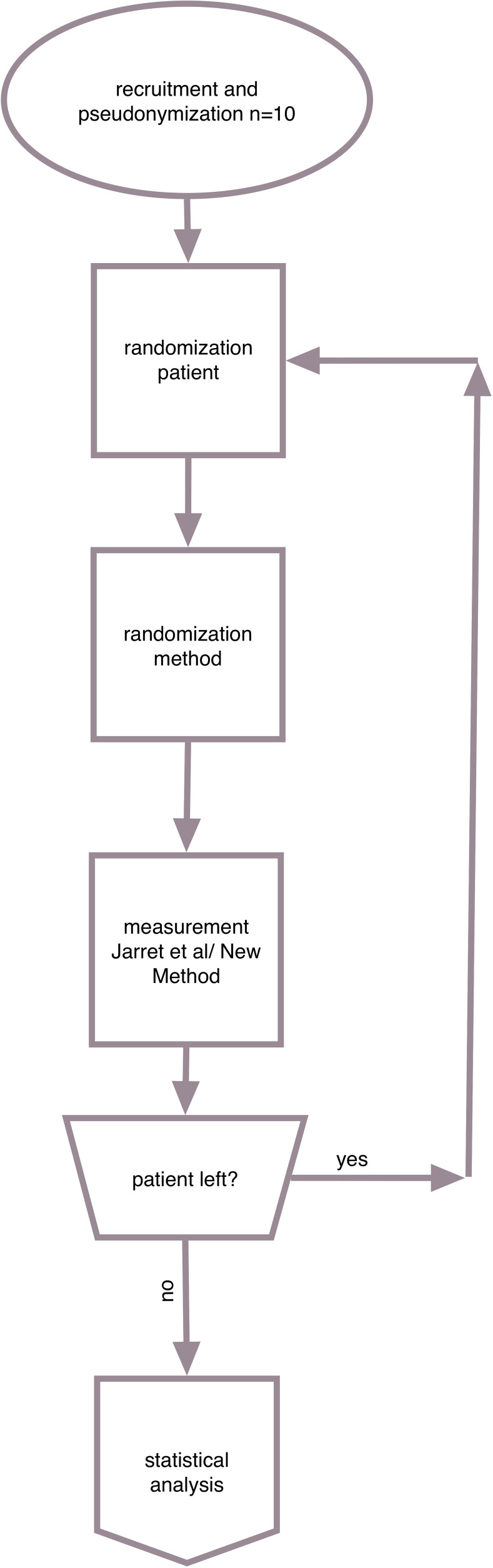


In this retrospective analysis, we performed 20 measurements on 10 patients (6 male and 4 female patients, mean age: 65 ± 23.31 years; median age: 43 years). The included patients were those admitted to our emergency department (Hannover Medical School, Hannover, Germany) between July 2008 and March 2020 and were treated according to generally accepted standards. The injuries were surgically treated with DHS/Screws (n = 8) or Intertrochanteric Nail (n = 2), and the procedures were performed by experienced trauma surgeons. The workflow we used is shown in Fig 1. Each patient was investigated by three experienced investigators(>6 years clinical experience) to assess the inter-observer-validity. All measurements were carried out with the program "Visage 7.1"(Visage Imaging, Berlin, Germany) and mediCAD (mediCAD Hectec GmbH, Landshut, Germany) by using a commercial personal computer (Windows 10 Professional Microsoft Corporation, One Microsoft Way, Redmond, WA USA). Statistical analysis was carried out with SPSS V27 (IBM, Armonk, NY, USA). The investigation was carried out on fully anonymized data.

Approval of the local ethics review board was obtained for our Study(Ethics Committee of Hannover Medical School). An assessment by the ethics committee and the in-house data protection officer is available. No concerns were raised. Informed consent was obtained in writing language.

**Fig 2:**
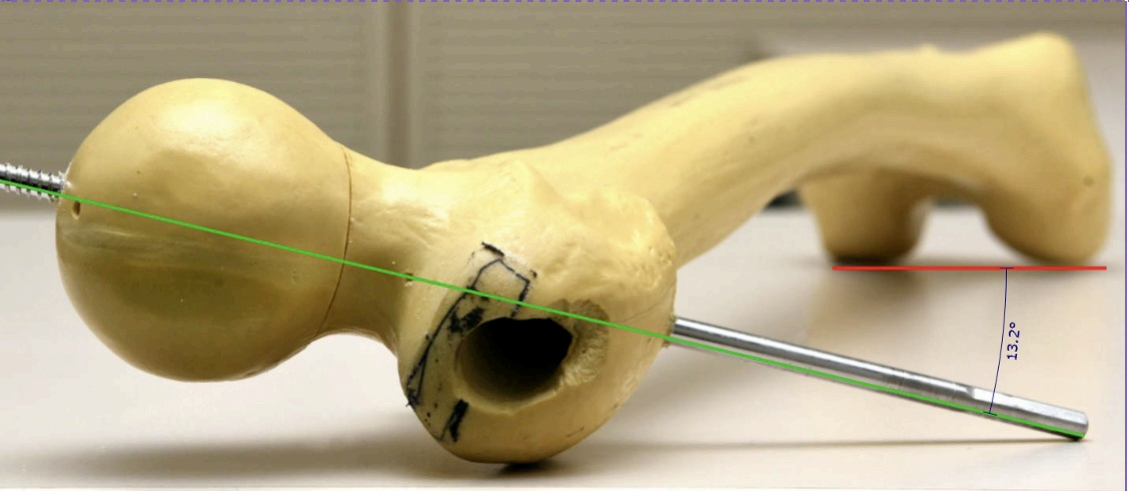
Undislocated SawBone: 13.2° antetorsion of the femoral neck.

**Fig 3:**
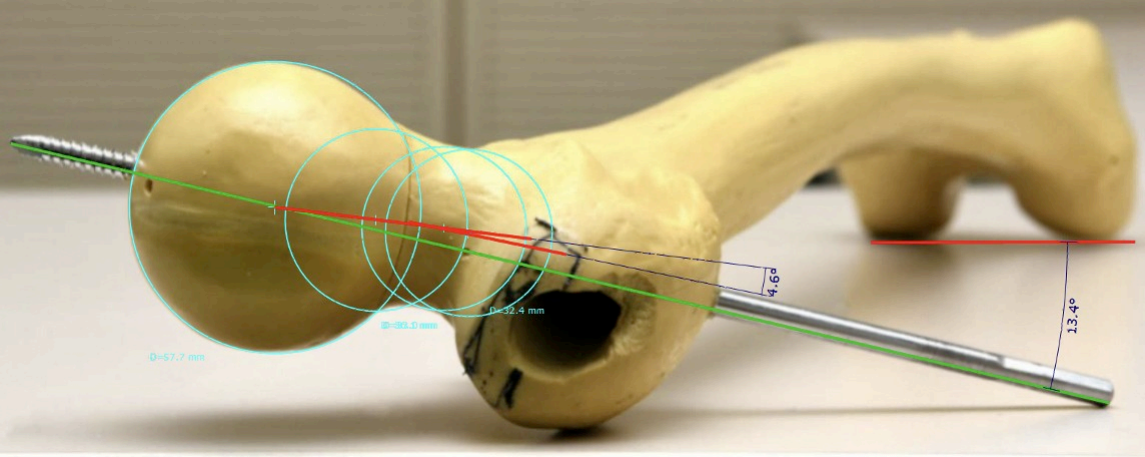
Undislocated SawBone (13.4° antetorsion of the femoral neck): Measurement shown without fracture (4.6° posterior axis deviation).

**Fig 4:**
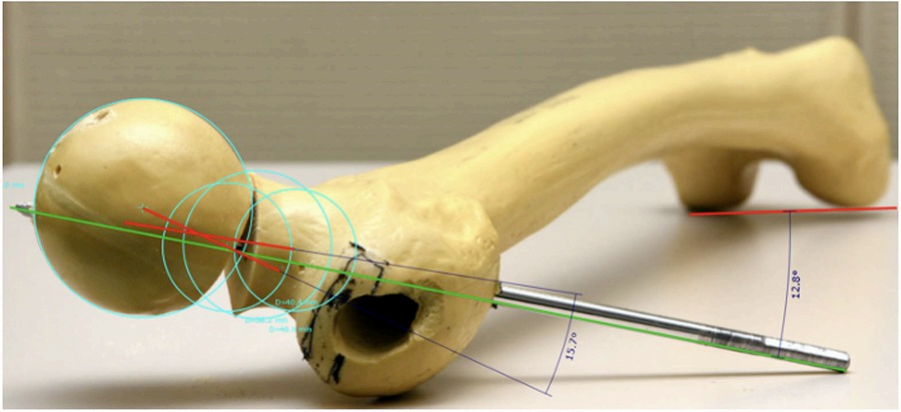
SawBone: 12.8° antetorsion of the femoral neck with an additional 15.7° anteversion.

**Fig 5:**
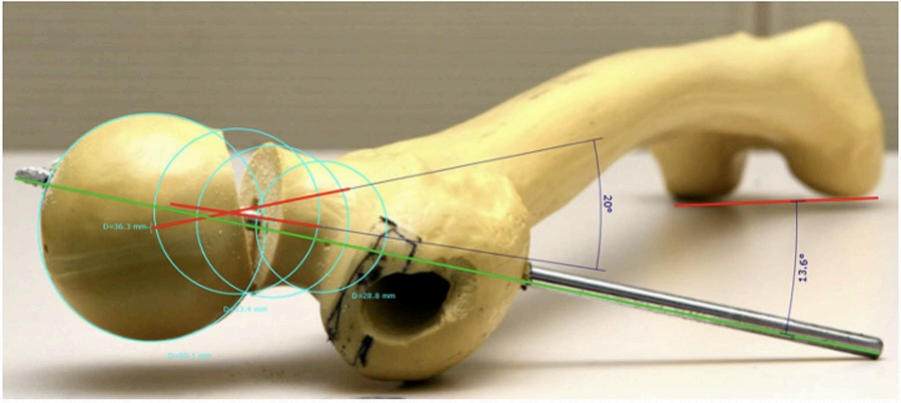
SawBone: 13.6° antetorsion of the femoral neck with an additional 20° retroversion.

To simplify the described problem and our proposed solution, the fracture and the measuring method are shown geometrically on a SawBone (Figs 2–5).

### Inclusion criteria

- Femoral neck fracture
- Age >18 years
- All sexes
- Treatment by osteosynthesis
- Postoperatively performed CT scan
- or Postoperatively performed CT angiography

### Exclusion criteria

- Serial injury of same long bone
- Pertrochanteric fractures
- Shaft fractures
- Treatment by arthroplasty
- Bilateral fractures

### CT scan

The postoperative CT scan was performed when clinical or radiographic hints of malrotation were noted. A standardized Torsion-Length-Difference CT scan (Somatom, Force, Siemens, Erlangen, Germany) was performed with the patient in the supine position and the limbs extended and mounted on a footrest to stabilize the position during the scan. CT cuts of 1.25-mm thickness were acquired through the femoral-neck region and through the condylar region of both femora simultaneously.

#### Method: Jarrett et al. [22]

**Fig 6:**
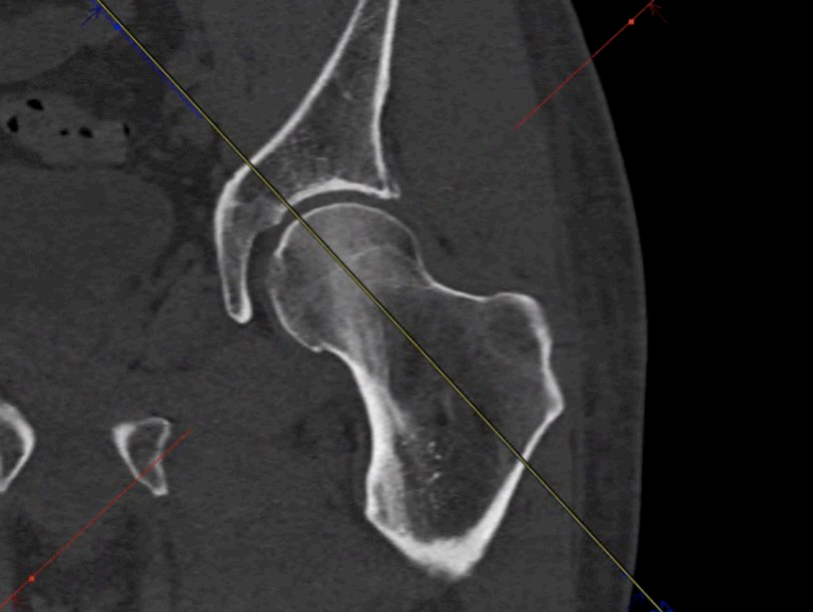
Setting the transversal section in coronal view.

Jarrett et al’s method [22] is usually applied to assess the malrotation of femoral fractures after long bone surgery. For the measurement, four sectional images are necessary. The layer thickness is set to 5 mm and at first, the femoral neck plane is set. The circumference of the femoral head and the femoral neck should be accessible in the same slice. For this purpose, the transverse section in the coronal view is aligned centrally along the femoral neck axis (Fig 6).

**Fig 7:**
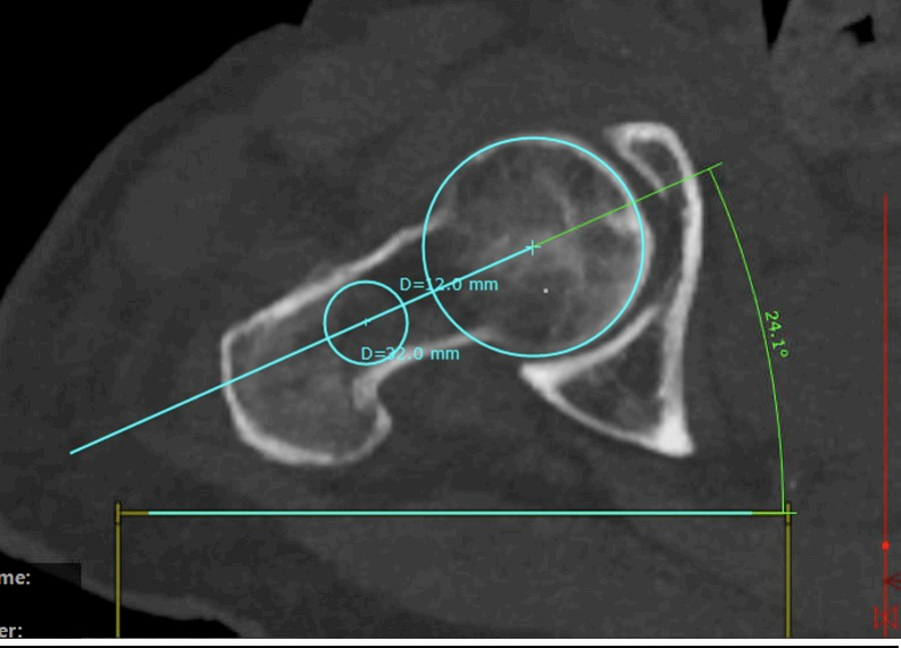
Middle of the femoral neck defined by connected circles (healthy side).

For the transversal section, a circle is fit to the femoral head and in the femoral neck, touching both cortices (Figs 7 and 9).

A straight line crossing the circle’s midpoints is drawn. The angle is measured to the zero level.

**Fig 8:**
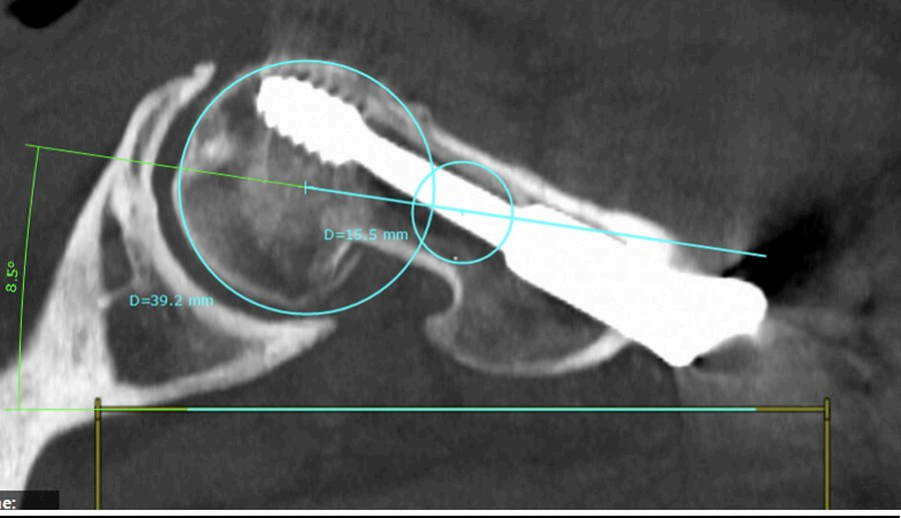
Middle of the femoral neck defined by connected circles (injured side).

**Fig 9:**
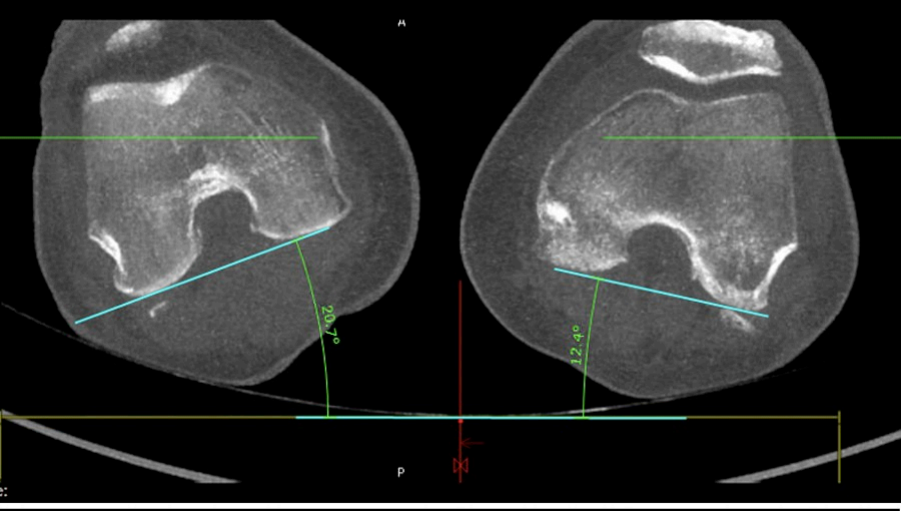
Setting angulation of femoral rotation at the condylar level.

Next, the condylar plane is set and a best fitting line at the dorsal edge of the condyles connecting both is drawn (Figs 8 and 9). The angle of this line is also referenced to the zero level.

By addition or subtracting, the antetorsion of the femoral neck in relation to the condylar plane can be defined.

### Method: New measurement

**Fig 10:**
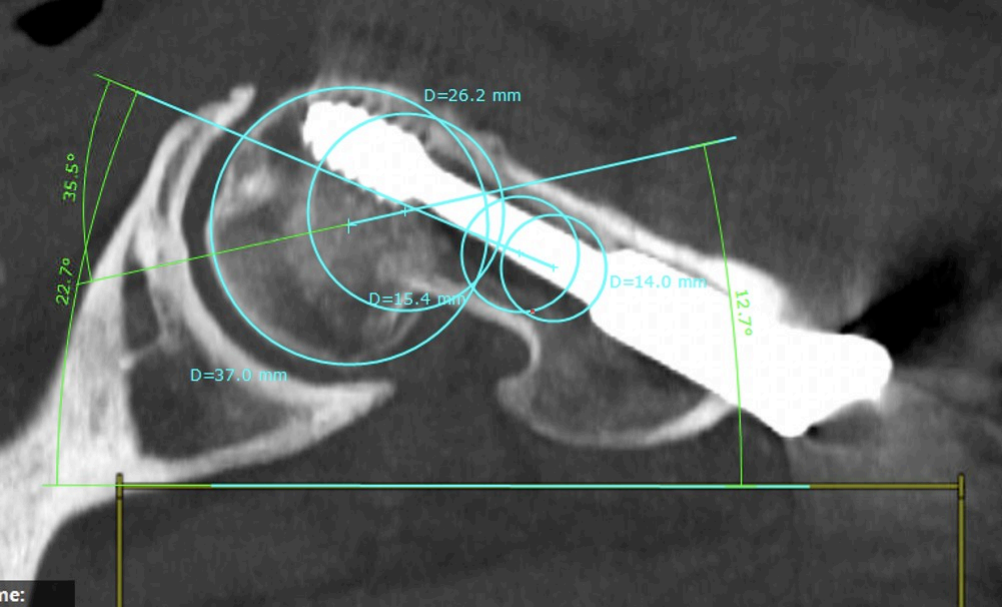
Defining the femoral neck by two circles and the orientation of the femoral head by two circles (injured side).

**Fig 11:**
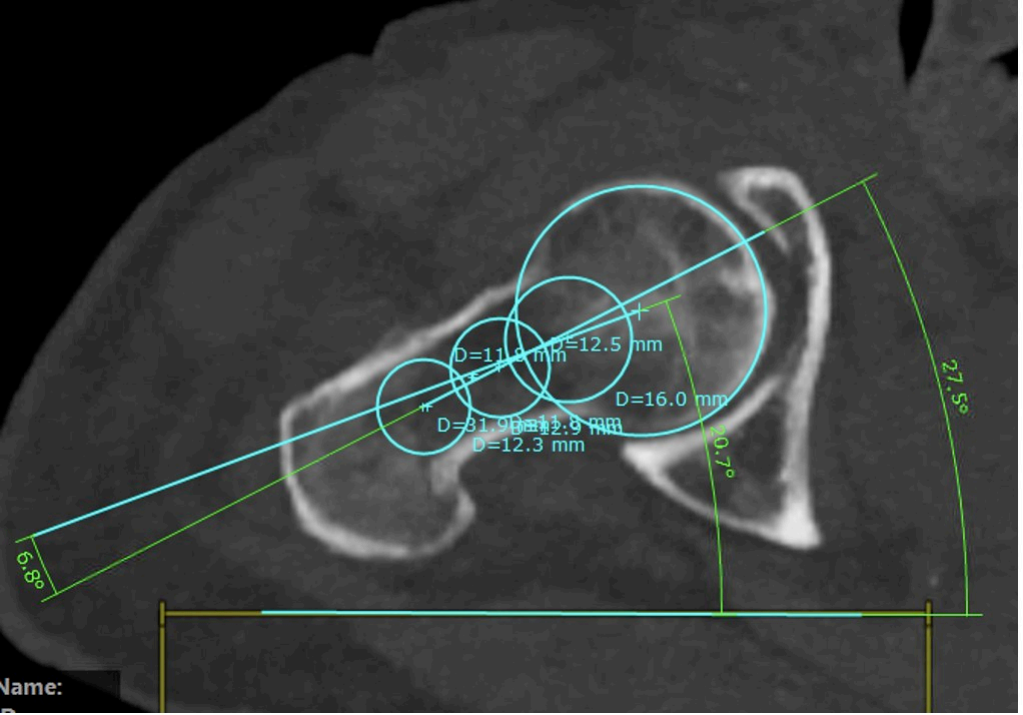
Defining the femoral neck by two circles and the orientation of the femoral head by two circles (healthy side).

For this technique, only two sectional images are necessary. We used a sectional thickness of 5 mm. Next, four circles were drawn—two for the medial and two for the lateral femoral neck fragment. Medially, the first circle should be best fitting to the femoral head. The second medial circle represents the opening of the femoral head towards the femoral neck. A line is drawn connecting both midpoints (Figs 10 and 11).

Next, two best fitting circles are drawn in the lateral fragment of the femoral neck. The circles should also touch both cortices. A second line is drawn connecting both lateral circles. The point of intersection marks the angle of malrotation (Figs 10 and 11).

A dorsal tilting of the medial axis is defined as a negative angle in the sense of a retroversion.

### Workflow

Every patient fitting the criteria was recruited and then pseudonymized by a number code. Three experienced physicians (two senior radiologists and a senior orthopaedic trauma surgeon) performed the measurements described above. Each patient was assessed by both investigators.

Both the patients and the order of the measurements were chosen randomly. Once a patient was chosen, the randomly chosen measurement technique was performed and stored in the data set. We performed only one of both measurements per selection. Next, another patient was chosen randomly for the above measurements. This process was continued till every included patient was measured by both techniques. The measurements were then merged in the data set using the pseudonym. Then, statistical analysis was performed. The workflow as described is shown in Fig 1.

### Statistical analysis

All statistical analyses were performed using Statistical Package for Social Sciences (SPSS) (SPSS 27.0, IBM Corp., Armonk, NY, USA). Normal distribution was analyzed by the Kolmogorov–Smirnov test. All values are presented in the form of mean±SD (standard deviation). Paired Student’s *t*-test was used to compare the mean difference between the applied methods. p<0.05 (two tailed) was considered to indicate statistical significance.

Statistical analysis was performed using a paired Student’s *t*-test (α = 0.05) to compare the mean difference between the applied methods. The reliability of investigators was assessed using intraclass correlation (ICC).

Qualitatively, the reliability is regarded as poor with ICC<0.5 and on the other hand, ICC > 0.8–0.9 is regarded as good or sometimes as excellent [23–25].

Intraobserver and interrater reliability was evaluated using the intraclass correlation (ICC). The two-way mixed model (absolute agreement) was used. The scoring system of Fleiss et al. [26] was utilized in our analysis (good: >0.75, fair: 0.4–0.75, and poor: <0.4). For intraobserver reliability the second assessment was performed after 3 Month.

## Results

First, we performed a descriptive analysis by examining data for age and sex distribution. We included 10 patients in total (6 men and 4 women, age range: 30–103 years) who fit the inclusion criteria. Out of these 10 patients one lateral femoral neck fracture with axial malrotation of the neck itself was shown.

### Analysis

The paired Student’s *t*-test was significant in 3 of 4 combinations. The direct comparison of the antetorsion between the standard method according to Jarrett et al. [22] and the direct new method presented here were significantly different in the results for the examined fractures (22.48° [95%CI: 17.62–27.33; p<0.001) and for the uninjured side (18.40° [95%CI: 12.47–24.32; p<0.001). Comparing the injured side with the healthy side, the new method shows a significant difference (-8.90° [95%CI: -17.50 to -0.30; p = 0.043]), whereas Jarrett et al’s. [22] method could not determine a significant difference between the injured and uninjured side in our measurements (-4.82° [95%CI: -12.77 to 3.13; p = 0.220]) (Table 1).

**Table 1:** Results of paired t-test between both methods combined.

	*Mean*	*Std*. *dev*	*Std*. *err*	*95%CI*	*p-value*
*AT Jarrett (inj)–AT new (inj)*	*22*.*48*	*10*.*37*	*2*.*32*	*17*.*62–27*.*33*	*<0*.*001*
*AT Jarrett (health)–AT new (health)*	*18*.*40*	*12*.*66*	*2*.*83*	*12*.*47–24*.*32*	*<0*.*001*
*AT Jarrett (inj)–AT Jarrett (health)*	*-4*.*82*	*16*.*98*	*3*.*80*	*-12*.*77–3*.*13*	*0*.*220*
*AT new (inj)–AT new (health)*	*-8*.*90*	*18*.*38*	*4*.*11*	*-17*.*50–0*.*30*	*0*.*043*

The intraclass correlation ICC is shown for the measured difference in rotation and the antetorsion separated by side, method, and between the different examiners is shown in Table 2.

**Table 2:** Results of ICC for interrater reliability Rater 1 and Rater 2 for torsional differences and antetorsion separated by side Jarrett et al. and new method.

	ICC	p-value	95%CI	Quality
Torsional difference Jarrett	0.801	0.012	0.199–0.951	good
Torsional difference new	0.935	<0.001	0.737–0.984	good
Right Jarrett	0.979	<0.001	0.914–0.995	good
Left Jarrett	0.966	<0.001	0.865–0.983	good
Right new	0.991	<0.001	0.965–0.998	good
Left new	0.935	<0.001	0.736–0.984	good

#### Rater 1 (Radiologist) and Rater 2 (Trauma Surgeon)

The ICC was 0.801 (good) (significance level: 95%CI, 0.199–0.951; p<0.012) for the torsion difference using Jarret et al’s method [22].

The novel method described above shows a comparable ICC of 0.935 (good) for the torsion difference (significance level: 95%CI: 0.737–0.984; p<0.001).

If the examinations, applying Jarret et al’s method [22], are separated according to the patients sides, our data show an ICC between the examiners on the right as 0.979 (good) (95%CI: 0.914–0.995; p<0.001) and on the left as 0.966 (good) (95%CI: 0.865–0.983; p<0.001), denoting high significance.

For comparison, the new method assumed an ICC of 0.991 on the right side (good) (95%CI: 0.965–0.998; p<0.001) and of 0.935 (good) (95%CI: 0.736–0.984; p<0.001) on the left.

#### Rater 1 (Radiologist) and Rater 3 (Radiologist)

As shown in Table 3 the ICC was 0.977(good) (95%CI: 0.908–0.994; p<0.001) for torsional difference in Jarret et al. [22] The new method in torsional difference shows ICC 0.851(95%CI: 0.399–0.963; p<0.005). In separation by side the method after Jarret er al. [22] shows right sided an ICC of 0.974 (95%CI: 0.895–0.993; p<0.001) and 0.927(95%CI: 0.704–0.982; p<0.001) respectively for the left side.

**Table 3:** Results of ICC for interrater reliability Rater 1 and Rater 3 for torsional differences and antetorsion separated by side Jarrett et al. and new method.

	ICC	p-value	95%CI	Quality
Torsional difference Jarrett	0.977	<0.001	0.908–0.994	good
Torsional difference new	0.851	<0.005	0.399–0.963	good
Right Jarrett	0.974	<0.001	0.895–0.993	good
Left Jarrett	0.927	<0.001	0.704–0.982	good
Right new	0.923	<0.001	0.691–0.981	good
Left new	0.846	<0.005	0,380–0.962	good

Compared to the novel method, it assumes an ICC 0.923(95%CI: 0.691–0.981; p<0.001) on the right side and on the left 0.846 (95%CI: 0,380–0.962; p = 0.005).

#### All raters

As shown in Table 4 the ICC was 0.887(good) (95%CI: 0.668–0.969; p<0.001) for torsional difference in Jarret et al. [22] The new method in torsional difference shows ICC 0.933(95%CI: 0.802–0.982; p<0.001). In separation by side the method after Jarret er al. [22] shows right sided an ICC of 0.978 (95%CI: 0.936–0.994; p<0.001) and 0.955(95%CI: 0.867–0.988; p<0.001) respectively for the left side.

**Table 4:** Results of ICC for interrater reliability for all raters combined for torsional differences and antetorsion separated by side (Jarrett et al. and novel method).

	ICC	p-value	95%CI	Quality
Torsional difference Jarrett	0.887	<0.001	0.668–0.969	good
Torsional difference new	0.933	<0.001	0.802–0.982	good
Right Jarrett	0.978	<0.001	0.936–0.994	good
Left Jarrett	0.955	<0.001	0.867–0.988	good
Right new	0.971	<0.001	0.914–0.992	good
Left new	0.910	<0.001	0,736–0.976	good

Compared to the novel method, it assumes an ICC 0.971(95%CI: 0.914–0.992; p<0.001) on the right side and on the left 0.910 (95%CI: 0,736–0.976; p = 0.001).

#### Intraobserver reliability (Trauma Surgeon)

The ICC for intraobserver reliability show different results for both techniques as seen in Table 5.

**Table 5:** Results of ICC for Intraobserver reliability for torsional differences and antetorsion separated by side Jarrett et al. and new method.

	ICC	p-value	95%CI	Quality
Torsional difference Jarrett	0.736	0.030	-0.063–0.934	fair
Torsional difference new	0.907	<0.001	0.977–10.792	good
Right Jarrett	0.919	<0.001	0.980–12.302	good
Left Jarrett	0.955	<0.001	0.989–22.255	good
Right new	0.866	<0.003	0.460–0.967	good
Left new	0.965	<0.001	0.991–28.822	good

For the method Jarrett et al. [22] we found an ICC 0,736(fair) (95%CI: -0.063–0.934; p = 0.030) for difference in Torsion.

For the new Method, the ICC for difference in rotation remains (good) 0.907 (95%CI: 0.977–10.792; p<0.001). Divided by side, the intraobserver reliability shows 0.919(good) (95%CI: 0.980–12.302; p<0.001) for the right performing Jarrett et al. [22] Respectively for the left the ICC is 0.955(good) (95%CI: 0.989–22.255; p<0.001).

When it comes to the new method, the results remains good with ICC 0.866(good) (95%CI: 0.460–0.967; p = 0.003) respectively 0.965(good) (95%CI: 0.991–28.822; p<0.001) for right and left. All results are highly significant.

## Discussion

For fractures of the long bone in the shaft area, various methods have been established since the introduction of CT to measure a postoperative incorrect torsion along the longitudinal axis [10, 12, 22] and to define the area from which clinical impairments are likely to originate [1, 8, 10, 11].

However, regardless of their wide use in femoral shaft fractures, the application of these methods is problematic in the region proximal to the trochanteric plane, because the measurement methods described do not work well in this region.

Similar to the systematic difference in the absolute results of the measurements with the established methods, attempts to measure postoperative misalignments after osteosynthesis of the femoral neck also show significant differences, depending on the examiner.

In our clinic, there was a consistent considerable variance in the postulated rotational errors between the examiners after osteosynthesis treatment of the femoral neck axis, owing to the application of one of the established methods. Yet, no method has been described to address this unique problem of malrotation after femoral neck fractures.

In this study, we present a type of measurement to reliably detect a malposition of the reduction after osteosynthesis.

For the radiological measurement of a rotational error, a special type of CT scan for measuring the torsional difference is essential. However, as this is not a standard diagnostic procedure after the surgical treatment of femoral neck fractures, the number of patients who are eligible for an evaluation is correspondingly low.

The interrater reliability was calculated for the combinations Trauma Surgeon/Radiologist respectively Radiologist/Radiologist and for all. In the examined cohort, we found an “excellent” interrater reliability for both combinations ICC = 0.979/0.974/0.978 for the right side and “excellent” values of ICC = 0.966/0.927/0.955 on the left, by using Jarrett et al’s method for determination of the antetorsion separately according to sides [22]

Separated by side, the new method shows comparable results with ICC values of 0.991/0.923/0.971 and 0.935/0.846/0.910 for the right and left sides, respectively. Due to the very high significance, Jarrett et al’s method [22] showed an excellent reliability, consistent with values reported in literature. In 2016, Kaiser et al. [10] reported ICC values ranging between 0.94 and 0.98, based on Jarrett et al’s method [22].

However, more important than the separately given antetorsion of femoral neck axis, because of a physiological variance depending on age and sex [27], is the assessment of the torsional difference of both sides, which is ultimately the clinical decision-making factor for or against surgical treatment. The interrater reliability of Jarrett et al’s method [22] and the new method was ICC = 0.801/0.977/0.887 and ICC = 0.935/0.851/0.933, respectively. Thus, the new method presented in this study is clearly superior.

When it comes to the intraobserver reliability, the measured cohort shows a significant better ICC for the novel method compared to Jarrett et al, with 0.907 respectively 0.786 for comparison in torsional differences. The good results compared with the fair findings in Jarrett et al’s Method, again show the superiority of the novel method.

Direct comparison of the resulting measurements showed a significant difference for three of the four method comparisons. Both, for the injured side with mean 22.48° and the uninjured side with a mean of 18.40°, Jarrett et al’s. [22] method differed considerably and significantly on average compared to the new method presented here (Table 1). This method dependency for the determination of the absolute values is in line with the literature, wherein systematic differences were noted in the absolute results between the methods used [10]. As the rotation errors from 15° are considered worthy of correction [1, 10, 11], an inter-method difference of >22° or >18° is not negligible.

Even if a systematic difference in the methods is consistent with the literature, similar values within an individual are expected when comparing two healthy sides, but different values when comparing the injured vs. healthy sides. Here, the results of this study concerning Jarrett et al’s method [22] show no significant differences between the healthy and injured side, within one individual with in mean -4.82° (95% CI -12.77 to 3.13; p <0.220) difference. These results show, in a collective where clear rotational errors were observed, the established methods, in this study exemplified by the method of Jarrett et al. [22], in this specific question of malrotation as a result of osteosynthesis in femoral neck fractures, does not seem to be a valid method. In contrast, the described new method makes a significant difference between the injured and healthy sides with a mean rotational difference of -8.90° (95%CI: -17.50 to -0.30; p = 0.043) (Table 1).

But our study has some limitation. This work describes how the new method works, delimits it from the established methods, and introduces it into the general discourse. However, this study does not provide any data on the validity of the new method. Even if the established methods are not yet validated for use in femoral neck fractures, we cannot provide data for validation on the new method either yet. Further, the sample size was small (n = 10), even though this number met the minimum requirements for an adequate calculation of the ICC. The low case count results from the fact that the necessary Torsion-Length-Difference CT scan causes high radiation and is not a standard method for assessing the position and reduction in the postoperative setting after femoral neck osteosynthesis. In addition, we excluded rotation influencing cofactors such as multi-level fractures or bilateral fractures. But still due to the multidimensional problem in femoral neck fractures, one patient in the examined cohort shows a combined dislocation. In addition to the dorsal rotational malalignment in relation to the femoral longitudinal axis, there was a slight dorsal rotational component in the longitudinal axis of the neck itself. This special form of rotation error in femoral neck fractures is the result of the force applied when screwing in the screw. This special form of incorrect rotation has the potential to imitate a dorsal or ventral tilting of the femoral neck [28, 29].

As shown, the established methods may fail to address this unique question of malrotation after femoral neck fractures. These methods were originally devised to assess torsional errors in the femoral shaft axis. Nevertheless, they are often used inappropriately for pertrochanteric and femoral neck fractures. However, the method presented here makes a significant difference between the injured and uninjured side and shows significant differences in results, when compared to conventional measurement methods. The interrater reliability determined in this study is excellent and even higher than that obtained with Jarret et al’s method in the assessment of rotational differences [22].

In addition, the method described here helps to reduce radiation exposure. Whereas the condyles also had to be irradiated for the established methods, only a narrow area from the head of the femoral neck including the greater trochanter is sufficient for the new method. This results in an extended applicability. A narrow area of the osteosynthesis can already be examined intraoperatively by CT scan, so that a dynamic response to possible rotational errors can be made.

We believe that appropriate validation in cadaver studies or using SawBones as the next step with a larger number of cases will further highlight the usefulness and impact of this method.

## Conclusion

The postoperative assessment of torsional errors after open reduction and internal fixation of femoral neck fractures is difficult and an objective measurement method has not yet been described.

We believe that the measurement method presented in this study is a useful tool to objectify the postoperative deformities in this area and make therapy recommendations in the future. Nevertheless, further investigations with larger numbers of cases and more independent investigators are necessary. In future studies, measurements should be validated in cadaver studies or using SawBones.

## Supporting information

S1 File (XLSX)

S2 File (XLSX)
